# Research Progress on the Mechanism and Management of Septic Cardiomyopathy: A Comprehensive Review

**DOI:** 10.1155/2023/8107336

**Published:** 2023-11-20

**Authors:** Xue-Bin Pei, Bo Liu

**Affiliations:** ^1^Emergency Medicine Clinical Research Center, Beijing Chao-Yang Hospital, Capital Medical University, Beijing 100020, China; ^2^Department of Emergency Medicine, Beijing YouAn Hospital, Capital Medical University, Beijing 100069, China

## Abstract

Sepsis is defined as a kind of life-threatening organ dysfunction due to a dysregulated host immune response to infection and is a leading cause of mortality in the intensive care unit. Sepsis-induced myocardial dysfunction, also called septic cardiomyopathy, is a common and serious complication in patients with sepsis, which may indicate a bad prognosis. Although efforts have been made to uncover the pathophysiology of septic cardiomyopathy, a number of uncertainties remain. This article sought to review available literature to summarize the existing knowledge on current diagnostic tools and biomarkers, pathogenesis, and treatments for septic cardiomyopathy.

## 1. Introduction

Sepsis is defined as a type of life-threatening organ dysfunction due to a dysregulated host immune response to infection and is a leading cause of death worldwide with a high mortality rate of nearly 30% [1, 2]. Multiple organ dysfunctions can result from systemic inflammation. Since the heart is quite vulnerable, myocardial dysfunction caused by sepsis, which is known as septic cardiomyopathy, is frequently reported. Septic cardiomyopathy was firstly observed in 1967 and initially defined in the 1980s [3–5]. Septic cardiomyopathy is characterized by acute ventricular dysfunction with impaired contractility, which can be reversed and restored within 7–10 days [6].

Several mechanisms involved in septic cardiomyopathy have been proposed, including inflammatory factors such as damage-associated molecular patterns (DAMPs), pathogen-associated molecular patterns (PAMPs), nitric oxide (NO), calcium handling, mitochondrial dysfunction, and complements. Emerging mediators, such as exosomes and noncoding RNAs (ncRNAs), including microRNAs (miRNAs) and long noncoding RNAs (lncRNAs), were recently demonstrated to contribute to the development of septic cardiomyopathy [7, 8]. There is no specific treatment for septic cardiomyopathy yet, mainly of which is symptomatic, supportive, and applied to treat the underlying sepsis. Understanding how septic cardiomyopathy occurs may help to provide benefits for patients with septic cardiomyopathy.

In this article, we mainly review and summarize the current literature concerning septic cardiomyopathy, with a focus on diagnostic tools, potential biomarkers, pathogenic mechanisms, and treatments for septic cardiomyopathy.

## 2. Epidemiology

The prevalence of septic cardiomyopathy varies greatly because of the diverse definitions used in articles (Table 1). A younger age, a positive etiological culture result, a history of diabetes, and a history of heart failure are reported to be risk factors for septic cardiomyopathy, and the occurrence of septic cardiomyopathy indicates a higher short-term mortality [11, 13].

## 3. Mechanism

**Figure 1:**
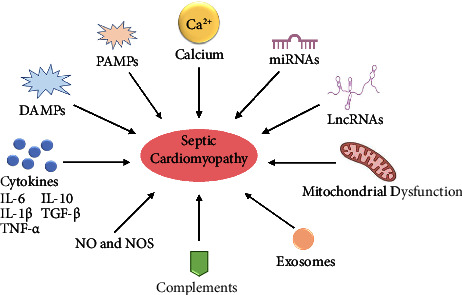
Pathophysiology of septic cardiomyopathy. This illustration shows the potential mechanisms contributed to septic cardiomyopathy. The pathways involved are the activation of inflammatory response including PAMPs, DAMPs, cytokines, NO, NOS, and complements; disorders of exosomes, miRNAs and lncRNAs; mitochondrial dysfunction; and the imbalance of calcium handling. PAMPs, pathogen-associated molecular patterns; DAMPs, damage-associated molecular patterns; NO, nitric oxide; NOS, nitric oxide synthase; LncRNA, long noncoding RNA.

At present, the pathophysiology of septic cardiomyopathy is not completely clear and is still under exploration. Studies focusing on septic cardiomyopathy have investigated several mechanisms, including inflammation, calcium handling, mitochondrial dysfunction, complements, exosomes, and ncRNAs as shown in Figure 1.

### 3.1. Inflammation

#### 3.1.1. PAMPs and DAMPs

Since sepsis is caused by a dysregulated immune response to infection, the inflammatory response should be involved in septic cardiomyopathy, driven by PAMPs and DAMPs [1]. Lipopolysaccharide (LPS), as the best-known type of PAMP molecule, has been a key component in the pathogenesis of septic cardiomyopathy. LPS can bind to pattern-recognition receptors and activate the transcription of inflammatory mediators, thus inducing myocyte apoptosis [20]. The mechanisms of cardiac depression by LPS also involve mitochondrial dysfunction, ion channel dysfunction, and calcium homeostasis alteration [21]. DAMPs, such as high-mobility group box 1 protein (HMGB1) and heat shock proteins (HSPs), are also essential regulators in septic cardiomyopathy. Previous studies have cited that HMGB1 secreted by viable cardiomyocytes may mediate the LPS-induced myocardial contractile dysfunction [22], and HMGB1 could lead to cardiac dysfunction by enhancing sarcoplasmic reticulum calcium leakage [23]. The HSP70 family was reported to play protective roles in septic cardiomyopathy by the maintenance of endothelial permeability and suppression of autophagy activation [24, 25].

#### 3.1.2. Cytokines

Cytokines are key mediators in inflammatory conditions like sepsis. Numerous studies have demonstrated that interleukin (IL)-6 has good performance in sepsis diagnosis and prognosis [26], but its role in the development of sepsis remains controversial. Some researchers have reported that IL-6 mediated cardiac inflammation and dysfunction in a burn plus sepsis model [27], and the inhibition or blockage of IL-6 was supposed to be a treatment for sepsis [28], while others suggested that IL-6 activates neutrophils to enhance the killing of bacteria to improve the survival rates of sepsis patients [29], and the loss of IL-6 signaling led to impaired monocyte/macrophage killing of pathogens, which could be reversed by IL-6 supplementation [30]. Like IL-6, IL-1*β* also has diverse roles in sepsis. It was reported that IL-1*β* protects against sepsis by activating the proliferation and differentiation of bone marrow cells into dendritic cells [31]. Meanwhile, in sepsis-induced cardiomyopathy, IL-1*β* contributed to myocardial dysfunction by inducing cardiac atrophy, impairing contraction and relaxation of cardiomyocytes, and boosting inflammatory cytokine expression levels [32]. Tumor necrosis factor (TNF)-*α*, as a major regulator of the inflammatory response, has been proposed to be a key contributing factor in septic cardiomyopathy. It was reported that the early depression of contractility was largely due to LPS-induced TNF-*α* synthesis [33], and TNF-*α* was suggested to be the core target of anti-inflammation in septic cardiomyopathy [34].

As well-known anti-inflammatory cytokines, IL-10 and transforming growth factor beta (TGF-*β*) act to modulate the inflammatory response during sepsis. IL-10, commonly produced by monocytes/macrophages and TH2 cells, was demonstrated to be increased and related to the outcome of sepsis [35, 36]; moreover, IL-10 exhibited both pro- and anti-inflammatory effects on innate and adaptive immunity in the septic environment [37]. TGF-*β*1 was revealed to block early cardiac depression induced by TNF-*α*, IL-1*β*, and septic serum in sepsis [38]. Growth differentiation factor 3, a member of the TGF-*β* family, was also implicated to reduce sepsis-induced cardiac dysfunction and mortality rates by altering macrophages to an anti-inflammatory phenotype [39].

### 3.2. Nitric Oxide (NO) and NO Synthase (NOS)

NO, which is produced from arginine by NOS, is a signaling molecule and acts as a regulator for cardiac functions in both normal and diseased hearts [40]. Studies have shown that both NO and NOS are involved in the pathogenesis of sepsis in several aspects, such as the maintenance of microcirculation homeostasis [41] and the regulation of vascular function [42]. In terms of myocardial dysfunction induced by sepsis, the roles of NO and NOS remain incompletely defined. There are some suggestions that the NO production is responsible for myocardial contractility maintenance in sepsis [43]. It was reported that myocardial NO levels increase after LPS treatment, and selective inhibition of NOS provides benefits for LPS-induced myocardial dysfunction; however, the decreased contractility was not necessarily related to the high content of myocardial NO [44]. Among the three isoforms of NOS, NOS2 and NOS3 are more relevant to sepsis. NOS2 was demonstrated to be required for endotoxin-induced cardiac impairment, but the deficiency of NOS2 failed to improve the mortality rate [45]. Overexpression of cardiomyocyte-specific NOS3 protected patients from myocardial depression in sepsis [46], while endothelial NOS3 might impair cardiac contractility in developing sepsis [47]. Taken together, it seems that NOS plays more important roles in the development of septic cardiomyopathy than NO.

### 3.3. Calcium Handling

It is common knowledge that the variation in intracellular calcium concentrations is a crucial regulator in cardiac myocyte function [48]. Calcium homeostasis has been reported as one of the underlying mechanisms of septic cardiomyopathy. It was reported that sepsis-induced myocardial dysfunction results from the impairment of sarcoplasmic calcium release, which is caused by blockade of the ryanodine channel [49], and prevention of sarcoplasmic reticulum calcium leakage by dantrolene could help to improve myocardial contractile dysfunction [50]. Moreover, in mice that survived the LPS challenge, the recovery of cardiac function correlate with the upregulation of calcium handling [51].

### 3.4. Mitochondrial Dysfunction

Mitochondria, which are abundant in cardiomyocytes, work as “power-factories” to supply energy to maintain the function of the heart. As such, mitochondrial dysfunction may lead to abnormal cardiomyocytes. It was found that there is an association between mitochondrial dysfunction, multiorgan failure, and poor outcome in septic patients [52], and improvements in mitochondrial function could contribute to biological function recovery [53].

There are several underlying mechanisms of mitochondrial dysfunction known to exist in septic cardiomyopathy [54]. Mitochondrial ultra-structural changes were observed in sepsis, including mitochondrial swelling, disruption of inner and outer membranes, formation of internal vesicles, and distortion of cristae [55–57]. Although morphological damage could be found in most models of sepsis, it was reported that myocardial or mitochondrial dysfunction might not relate to the observed morphological changes [55, 58].

Reactive oxygen species (ROS) are primarily generated by mitochondria and can be balanced under physiological conditions. In sepsis, however, the formation and clearance of ROS are imbalanced, with an accumulation of ROS and oxidative stress [54, 59]. Oxidative stress has been well recognized as a main regulator of mitochondrial dysfunction by impairing mitochondrial DNA, damaging myocardial structures, and causing cardiac dysfunction [54, 57, 60]. To explain the increased amount of ROS and oxidative stress in sepsis, NADPH oxidase 2 (NOX2) seems to be of interest. In cardiomyocytes isolated from LPS-induced sepsis models, increased oxidative stress, abnormal calcium transients, and decreased contractility were observed, and administration of NOX2 inhibitors diminished the abnormalities [61]. Mitochondria can also generate NO through the activation of mitochondrial NOS (mtNOS). Although several studies have reported that the overexpression of NO and mtNOS could lead to myocardial depression, the role of NO in septic cardiomyopathy remains controversial [40, 52].

During sepsis, changes in the inner mitochondrial membrane permeability will force the mitochondrial permeability transition pore (mPTP) to open, which can lead to mitochondrial dysfunction by triggering mitochondrial depolarization, respiratory inhibition, depression of oxidative phosphorylation, calcium release, and matrix swelling [62]. Previous studies have reported that the inhibition of mPTP by the administration of cyclosporine reduced multiorgan dysfunction and mortality rates in sepsis, thus providing a new therapy for septic cardiomyopathy [62, 63].

To maintain the essential function of mitochondria, a series of processes, including biogenesis, fission, fusion, and mitophagy are undoubtfully important. The fission and fusion processes work to maintain the number, size, shape, and biological characteristics of mitochondria. Mitochondrial structures together with fusion/fission processes were observed in LPS-treated animals at 24 h [55]. Fusion-to-fission imbalance was proved to be related to the progression of sepsis. In sepsis models of endotoxemia and cecal ligation and puncture (CLP), mitochondrial fusion and fission were found to be abnormal, and application of the fission inhibitor could lessen mitochondrial dysfunction [64].

### 3.5. Complements

The complement system is activated in sepsis, and the complement component 5 (C5a) has been reported to be strongly related with multiorgan failure during sepsis [65, 66]. The role of C5a in septic cardiomyopathy can be explained by two different mechanisms. First, C5a can change the concentration of calcium and ROS in cardiomyocytes, leading to cardiac dysfunction [67, 68]. Second, C5a can trigger the activation of MAPKs and Akt in cardiac myocytes and blockage of this activation response by a p38 inhibitor may attenuate the progression of cardiac dysfunction [69].

### 3.6. Exosomes

Exosomes are key regulators in various immunoregulatory functions of both donor and recipient cells due to their ability to deliver biological information to other cells, and they have drawn great attention recently [8]. It is reported that platelet-derived exosomes can induce vascular and myocardial dysfunction in septic patients [70, 71]. Furthermore, exosomes containing functional miR-223 were reported to play cardioprotective role in polymicrobial sepsis [72]. In view of the effects of exosomes, it may provide promising therapy for septic cardiomyopathy.

### 3.7. miRNAs and LncRNAs

Evidence has revealed that miRNAs and lncRNAs participate in the onset and development of septic cardiomyopathy. Several miRNAs, such as miR-125b, miR-150-5p, the miR-29 family, and the miR-30 family, were reported to be implicated in septic cardiomyopathy [73–75]. The lncRNA MIAT was reported to promote inflammatory response and oxidative stress in LPS-induced myocardial dysfunction [76]. Overexpression of the lncRNA SOX2OT could regulate mitochondrial function in mice with septic cardiomyopathy [77]. The lncRNA ZFAS1 promoted septic cardiomyopathy by mediating cardiomyocytes apoptosis [78]. These ncRNAs may provide novel insights into the diagnosis and treatment of sepsis-induced cardiac dysfunction.

## 4. Diagnosis

Septic cardiomyopathy is a severe complication of cardiac dysfunction due to systemic infection. To date, there are no international diagnostic criteria for septic cardiomyopathy. Here, we will discuss the use of measures depending on echocardiography (Table 2), serum biomarkers, and hemodynamic monitoring devices in the diagnosis of septic cardiomyopathy.

### 4.1. Echocardiography

#### 4.1.1. Left Ventricular (LV) Dysfunction

Echocardiographic variables are used to provide information on abnormalities in cardiac function. Among the many parameters, the LV ejection fraction (EF) (LVEF) is a fundamental parameter used to assess LV function [79]. Depressed LVEF and ventricular dilatation were introduced, and LV dilatation with a rise in end-diastolic diameter was also reported in patients with sepsis [3, 10, 80]. However, the concept of LV dilatation was questioned, and LV dilatation was failed to be detected in patients with septic shock [81]. Although an LVEF <40%–50% is commonly used as a diagnostic criterion for septic cardiomyopathy in many clinical studies [18, 82, 83], this parameter may not be the best indicator of LV systolic function because it changes in relation to loading conditions [84].

Mitral annular plane systolic excursion (MAPSE) has been suggested as a simple and sensitive parameter for the assessment of the global longitudinal function of the LV [85]. It is reported that a MAPSE of <11.65 mm may indicate septic cardiomyopathy with a sensitivity of 85.2% and a specificity of 70.7% [19], and a decreased MAPSE was a fine predictor of mortality in patients with septic cardiomyopathy [86]. Currently, global longitudinal strain (GLS) seems to be more reliable for the assessment of LV systolic function than LVEF, and a worse GLS value may predict a higher mortality rate in patients with sepsis [87, 88]. However, due to the requirement for high frame rate and image quality, the feasibility of GLS during septic shock was relatively low (42% for GLS vs 97% for LVEF) [89, 90]. It was recommended that LVEF could not be replaced by GLS, and these two parameters are complementary and should be used together [79].

LV diastolic dysfunction also happens in patients with sepsis. Early mitral annular velocity (e′) and transmitral early filling velocity/early mitral annular velocity (E/*e*′) are the most significant variables used to predict LV diastolic dysfunction; both e′ and E/*e*′ are independent and sound predictors of early mortality in patients with sepsis [12, 91, 92]. Another clinical study found that LV diastolic dysfunction correlated with the levels of N-terminal pro-B-type natriuretic peptide (NT pro-BNP) in critically ill patients with normal EF values [93]. It is unknown, however, whether diastolic dysfunction could be used to define septic myocardiopathy.

#### 4.1.2. Right Ventricular (RV) Dysfunction

Patients with sepsis also suffer from RV dysfunction characterized by decreased EFs and ventricular dilation [16, 94]. Tricuspid annular systolic excursion (TAPSE) is a measure of RV longitudinal function, and TAPSE <17 mm is generally an indicator of RV systolic dysfunction [95]. Several clinical studies have reported that RV dysfunction is linked to the severity of illness and a high mortality rate in sepsis [10, 96]. However, a meta-analysis failed to find the relationship between RV dysfunction and survival rate [97]. More research needs to be conducted to discern the true value of RV dysfunction in septic myocardiopathy.

Echocardiography may underestimate cardiac impairment because it fails to take the reduction of afterload into consideration. For this reason, the parameter “afterload-related cardiac performance” (ACP) was developed and described as the ratio between measured cardiac output (CO) and predicted normal CO at a given systemic vascular resistance; it quantifies the degree of cardiac impairment, and low ACP values indicate a poor prognosis in sepsis [98, 99]. However, as systemic vascular resistance was calculated from three parameters, namely, CO, mean arterial pressure, and central venous pressure, any deviations are expected to influence the accuracy.

### 4.2. Serum Biomarkers

#### 4.2.1. BNP and NT-proBNP

BNP and NT-proBNP are peptides released by cardiomyocytes in response to wall stretch and LV filling pressure [100, 101]. Studies have shown that plasma BNP and NT-proBNP concentrations are significantly elevated in patients with sepsis and might indicate myocardial dysfunction [102, 103]. However, it is unknown whether they can serve as reasonable discriminators of poor prognosis and filling pressure; it seems that the rise in BNP and NT-proBNP mainly occurred due to illness severity rather than septic cardiomyopathy [104, 105].

#### 4.2.2. Cardiac Troponin T (cTnT) and Troponin I (cTnI)

Cardiac troponin is a regulatory protein released following irreversible damage of myocardial cells, which can be detected in various conditions, such as acute coronary syndrome, heart failure, sepsis, myocarditis, pulmonary embolism, renal dysfunction, and acute neurological events [106]. During sepsis, elevated levels of circulating troponin were a sign of a heightened risk of death [107]. The mechanisms of the increased troponin TNI and TnT are multifactorial, such as the increased permeability of myocytes in response to inflammation, increased wall stress due to pressure or volume overload, cardiac toxicity by excessive catecholamines, and renal failure [20, 108, 109]. Although BNP and troponin levels were proven to be meaningful values in septic cardiomyopathy, none of them are considered specific for its diagnosis.

Researchers are making arduous efforts to seek new clues concerning septic cardiomyopathy. Proinflammatory cytokines, including IL-6, IL-1*β*, and TNF-*α*, are enriched in the serum of patients with septic cardiomyopathy, and the combined detection of these three factors provides both diagnostic and prognostic values for septic cardiomyopathy [110]. Besides, circulating histones were found to be a new mediator in cardiomyocyte injury in patients with sepsis [111]. Heart-type fatty acid-binding protein, a well-known cytoplasmic protein and cardiac biomarker, has been reported to be helpful in the recognition of myocardial damage and the prediction of 28-day mortality [112, 113]. Furthermore, gene-expression profiling revealed that *CCL2*, *STAT3*, *MYC*, *SERPINE1*, miR-29, and miR-30 families are closely related with septic cardiomyopathy [114]. This association needs to be proven by more clinical research, which may unveil potential biomarkers and clues for diagnosing septic cardiomyopathy.

### 4.3. Hemodynamic Monitoring Devices

The measurement of CO and other hemodynamic parameters is particularly important in septic cardiomyopathy. Pulmonary artery catheter (PAC) was previously used for hemodynamic monitoring of critically ill patients. However, the use of PAC has decreased since this device provides no benefits in patients' mortality [115]. Transpulmonary thermodilution method has been suggested to be an alternative to the PAC to measure CO and parameters of cardiac performance such as the cardiac function index (CFI) and global ejection fraction (GEF). It was reported that a low CFI and GEF obtained by transpulmonary thermodilution identified cardiac dysfunction in patients with sepsis [116]. Other devices such as pulse contour analysis were reliable for a continuous CO measurement in sepsis [117]. Furthermore, their use in diagnosis of septic cardiomyopathy needs to be verified.

## 5. Treatments

There are no standard practices for the treatment of septic cardiomyopathy yet. Patients with septic cardiomyopathy may benefit from well-established therapeutic approaches for sepsis and septic shock, which include the management of infection and the optimization of hemodynamics by fluid resuscitation and vasoactive medications [82, 118, 119]. Treatment strategies for septic cardiomyopathy are summarized in Table 3.

### 5.1. Drug Therapy

It is recommended that treatment of septic cardiomyopathy should be based on evidence of deficient organ perfusion, aiming at raising CO to an adequate level [120]. Fluid resuscitation is a fundamental strategy, and an initial administration of 20 ml/kg of balanced crystalloid is recommended to improve septic hypoperfusion, oxygen delivery, and organ function by elevating CO [119, 121]. However, excessive fluid volumes after initial resuscitation may result in an increase in cardiac filling pressure and tissue edema, indicating higher mortality rates [122, 123]; therefore, the hemodynamics status should be continuously monitored to assess the fluid responsiveness, thus guiding fluid therapy [124]. Approaches to determine fluid responsiveness include pulse pressure variation and systolic pressure variation assessed by arterial waveform, stroke volume variation by pulse contour analysis, LV end-diastolic area by echocardiography, global end-diastolic volume by a transpulmonary thermodilution, and central venous pressure by central venous catheter [125].

Norepinephrine is the first-line vasopressor to reverse hypotension in patients with sepsis due to its vasoconstrictive effects [119]. With a stronger *α*-adrenergic effect compared with *β*-1, norepinephrine increases afterload more than myocardial function, which may decrease CO and “unmask” cardiac dysfunction. It was reported that phenylephrine could inhibit cardiomyocyte apoptosis, thus suppressing cardiac dysfunction in septic mice, suggesting that phenylephrine may be beneficial in septic cardiomyopathy [126]. However, the risk of isolated *α*-vasoconstriction without *β*-1 may result in increased afterload, decreased CO, and worsen hemodynamics [6, 20].

Dobutamine is demonstrated to be the preferred choice of inotropic drugs for patients with persistent insufficient CO, despite adequate LV filling pressure or fluid loading [119]. However, the impact of dobutamine on septic cardiomyopathy is ambiguous. It was reported that the administration of dobutamine may increase cardiac index, heart rate, and LVEF in septic shock patients [127]. While recent research showed that dobutamine treatment could improve survival in septic rats with myocardial dysfunction, without recovering myocardial function and improving hemodynamics at the later stage of sepsis [128].

Investigations into other inotropic agents have also been conducted. Levosimendan, as an inotropic calcium sensitizer, has been reported to increase CO and systemic hemodynamics with a minimal increase in oxygen consumption in sepsis and septic myocardial depression [129–131]. However, the use of levosimendan did not lead to better performance in terms of facilitating less severe organ dysfunction or lower mortality rates among patients with cardiac dysfunction [132–134].

Tachycardia may result in increased oxygen consumption and reduce diastolic filling, making the use of *β*-blockers a potential therapy for septic cardiomyopathy [120, 135, 136]. A randomized controlled trial has revealed that the application of esmolol to lower heart rate improved outcome without impairing myocardial contractility and worsening hemodynamics in patients with septic cardiomyopathy [137]. However, a recent study involving 126 septic patients with tachycardia treated with continuous norepinephrine has revealed that the use of landiolol for managing tachycardia failed to reduce organ failure [138]. It should also be aware that the hemodynamics of patients with sepsis are unstable, and the use of *β*-blockers may aggravate hemodynamic instability due to its negative inotropic effect on myocardium.

The vitamin C protocol has been recently explored in sepsis, and its effect is ambiguous. Several clinical trials reported that the use of the vitamin C protocol did not significantly improve the outcome of patients with sepsis [139]. Other evidence supports that the effectiveness of the vitamin C protocol depends on the sepsis subphenotype, with the hyperinflammatory phenotype correlating with a better clinical result [140]. As for patients with septic cardiomyopathy, there is a clinical study demonstrating that the early application of the vitamin C protocol may provide benefits in terms of improving organ function and reducing mortality [141].

### 5.2. Mechanical Support

Mechanical therapy was also trialed in septic cardiomyopathy. As an effective tool in common stress-induced cardiomyopathy, intra-aortic balloon pumping (IABP) was reported to be beneficial in an animal model of septic shock with low cardiac index values [142]. Moreover, clinical studies including two contrasting cases of septic cardiomyopathy demonstrated that IABP was only effective in one case [143]. Separate retrospective clinical studies revealed that extracorporeal membrane oxygenation (ECMO) was a feasible treatment for patients with septic cardiomyopathy [144, 145]. Early initiation of ECMO for refractory shock due to septic cardiomyopathy not responding to medical management has been shown to have a mortality benefit with survival as high as 50%–70% [144, 146]. Polymyxin-B hemoperfusion to remove endotoxin has also been reported to be an option for septic cardiomyopathy [143]. An observational study has revealed that although the endotoxin activity was not related with septic cardiomyopathy, endotoxin removal by Polymyxin-B hemoperfusion was associated with recovery from septic cardiomyopathy [147]. Since IABP, ECMO, and Polymyxin-B hemoperfusion are all invasive strategies that can cause severe complications, more research is needed to better evaluate their value in septic cardiomyopathy.

## 6. Future Directions

Although the indications for treatment of septic cardiomyopathy are clinically based on maintaining sufficient organ perfusion, more thoughtful and careful investigations into therapeutic strategies of septic cardiomyopathy are warranted. It should be noted that cardiac performance dynamically changes due to hemodynamic alterations. Efforts have been made to characterize cardiovascular phenotypes in patients with sepsis, and five different profiles were identified using clinical and echocardiographic data, indicating theoretical ways to optimize cardiac function [148]. Therefore, understanding the heterogeneity of the cardiac response to sepsis may provide a more individual approach to care.

## 7. Conclusions

Although septic cardiomyopathy has been well recognized by its high incidence and mortality rates, difficulties in diagnosis and treatment remain unsolved. Echocardiography is currently the basic diagnostic method for myocardial depression in sepsis. Elevations in biomarkers such as BNP and TNI found in patients with septic cardiomyopathy also show potential value in diagnosis. Exploration of the pathogenic mechanisms could additionally provide novel insights into the treatment of septic cardiomyopathy. However, due to the lack of a standard definition of septic cardiomyopathy, the findings reported in different studies may be diverse and able to be challenged. More clinical studies are still needed to better understand septic cardiomyopathy.

## Figures and Tables

**Table 1:** Recent studies of septic cardiomyopathy.

References	Populations	Time of echocardiography	Results
Bouhemad et al. [9]	45	On days 1, 2, 3, 4, 7, and 10	18% with a reversible increase in cTnI and LVEF < 50%, 18% with a reversible increase in cTnI, and impairment of LV relaxation
Furian et al. [10]	45	Within the first 24 h, again 72 h, and 7 days after admission	33% with LVEF < 55%, and 30% with RV tissue Doppler peak systolic velocity <12 cm/s
Hanumanth et al. [11]	359	Within 72 h of admission, 6 months prior to admission or within 3 months after the diagnosis of sepsis	5.3% with a new onset decline in LVEF ≤ 50%, or ≥ 10% decline in LVEF compared to baseline in patients with reduced EF
Landesberg et al. [12]	262	On the day after admission to ICU, and the next day	23.3% with LVEF ≤ 50%, and 50% with diastolic dysfunction
Liang et al. [13]	3530		28.2% with LVEF < 50%, or with global LV hypokinesis or LV systolic dysfunction
Lu et al. [14]	100	Within 24 h of admission to ICU	21% with LV systolic dysfunction, 40% with LV diastolic dysfunction, and 12% with RV systolic dysfunction
Orde et al. [15]	60	Within 24 h of meeting sepsis criteria	32% with RV dysfunction, 20% with LV dysfunction, and 17% with both LV and RV dysfunction
Pulido et al. [16]	106	Within 24 h, at day 5 or day of dismissal of ICU	37% with LV diastolic dysfunction, 27% with LV systolic dysfunction, and 31% with RV dysfunction
Song et al. [17]	342	Within 48 h after ICU admission	14.3% with LVEF < 50% or ≥ 10% decrease in baseline EF that recovered within 2 weeks
Sato et al. [18]	210	Within 24 h of admission	13.8% with LVEF < 50% or *a* ≥ 10% decrease compared to the baseline EF which recovered within 2 weeks
Song et al. [19]	143	Within 24 h after admission to ICU	18.2% with LVEF < 50%

LV, left ventricular; LVEF, LV ejection fraction; RV, right ventricular; cTnI, cardiac troponin I; ICU, intensive care unit.

**Table 2:** Echocardiography parameters of septic cardiomyopathy.

Variables	Thresholds	Strengths	Limitations
LVEF	LVEF < 40%–50%	Physician familiarity, easy to obtain	LVEF depends on loading conditions, quantification varies by “eyeballing”
MAPSE	MAPSE < 11.65 mm	Easy to obtain	Preload and angle dependent, fail to detect regional myocardial abnormalities, vary due to cardiac size
GLS	GLS < −18%–−20%	Independent of loading conditions and angle, more sensitive and less varied	Low feasibility, lack of consensus on thresholds for abnormal values
LV diastolic dysfunction	e′ < 7 cm/s (septal) or <10 cm/s (lateral); E/*e*′ > 14	Easy to obtain	May be affected by regional wall motion abnormalities and patients' age
RV dysfunction	TAPSE < 17 cm	Easy to obtain	May be affected by LV dysfunction and tricuspid regurgitation

LVEF, left ventricular ejection fraction; MAPSE, mitral annular plane systolic excursion; GLS, global longitudinal strain; *e*′, early mitral annular velocity; E, transmitral early filling velocity; TAPSE, tricuspid annular systolic excursion.

**Table 3:** Treatment strategy of septic cardiomyopathy.

Treatment strategy		Benefits	Problems
Noninvasive	Fluid resuscitation	Increasing preload may elevate cardiac output	Excessive fluid may lead to higher mortality
	Vasopressors	May reverse hypotension due to the vasoconstrictive effects	Increased afterload may “unmask” cardiac dysfunction
	Dobutamine	May improve cardiac parameters	Risk of tachycardia, impact on outcome is ambiguous
	Levosimendan	May increase cardiac output and systemic hemodynamics	No change on mortality
	*β*-blockers	May decrease myocardial demand and improve outcome	Negative inotropic effect, may aggravate hemodynamic instability
	Vitamin C protocol	May improve organ function and outcome	More clinical research is needed

Invasive	IABP	May provide supportive care and mortality benefits	Invasive, may cause severe complications
	ECMO		
	Polymyxin-B hemoperfusion		

IABP, intra-aortic balloon pumping; ECMO, extracorporeal membrane oxygenation.
